# A second monoclinic polymorph of 3,5-di-*tert*-butyl-2-hy­droxy­benzaldehyde

**DOI:** 10.1107/S1600536813022010

**Published:** 2013-08-14

**Authors:** Seik Weng Ng

**Affiliations:** a Department of Chemistry, University of Malaya, 50603 Kuala Lumpur, Malaysia; b Chemistry Department, Faculty of Science, King Abdulaziz University, PO Box 80203 Jeddah, Saudi Arabia

## Abstract

In the title mol­ecule, C_15_H_22_O_2_, there is an intra­molecular hydrogen bond involving the hy­droxy and aldehyde groups and forming an *S*(6) ring. The mean plane of the non-H atoms of this ring [(H)O—C C—C=O, with a maximum deviation of 0.013 (1) Å] are essentially coplanar with the benzene ring, forming a dihedral angle of 2.29 (8)°.

## Related literature

For a monoclinic polymorph which contains two independent mol­ecules in the asymmetric unit, see: Chu *et al.* (2004 ▶); Ng (2013 ▶); Tooke & Spek (2004 ▶).
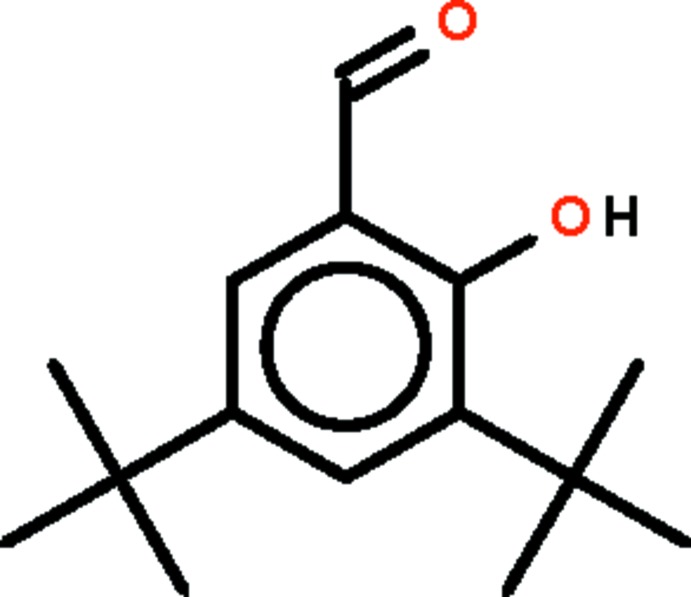


## Experimental

### 

#### Crystal data

C_15_H_22_O_2_
*M* *_r_* = 234.33
Monoclinic, 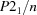

*a* = 9.8347 (6) Å
*b* = 11.1775 (5) Å
*c* = 13.1287 (8) Å
β = 110.614 (7)°
*V* = 1350.80 (13) Å^3^
*Z* = 4
Mo *K*α radiation
μ = 0.07 mm^−1^
*T* = 100 K
0.40 × 0.30 × 0.20 mm

#### Data collection

Agilent SuperNova Dual diffractometer with an Atlas detector
Absorption correction: multi-scan (*CrysAlis PRO*; Agilent, 2013 ▶) *T*_min_ = 0.971, *T*_max_ = 0.985
7536 measured reflections
3130 independent reflections
2511 reflections with *I* > 2σ(*I*)
*R*_int_ = 0.027

#### Refinement

*R*[*F* ^2^ > 2σ(*F* ^2^)] = 0.046
*wR*(*F* ^2^) = 0.119
*S* = 1.02
3130 reflections
158 parameters
H atoms treated by a mixture of independent and constrained refinement
Δρ_max_ = 0.29 e Å^−3^
Δρ_min_ = −0.23 e Å^−3^

### 

Data collection: *CrysAlis PRO* (Agilent, 2013 ▶); cell refinement: *CrysAlis PRO*; data reduction: *CrysAlis PRO*; program(s) used to solve structure: *SHELXS97* (Sheldrick, 2008 ▶); program(s) used to refine structure: *SHELXL97* (Sheldrick, 2008 ▶); molecular graphics: *X-SEED* (Barbour, 2001 ▶); software used to prepare material for publication: *publCIF* (Westrip, 2010 ▶).

## Supplementary Material

Crystal structure: contains datablock(s) global, I. DOI: 10.1107/S1600536813022010/lh5640sup1.cif

Structure factors: contains datablock(s) I. DOI: 10.1107/S1600536813022010/lh5640Isup2.hkl

Supplementary material file. DOI: 10.1107/S1600536813022010/lh5640Isup3.cml

Additional supplementary materials:  crystallographic information; 3D view; checkCIF report

## Figures and Tables

**Table 1:** Hydrogen-bond geometry (Å, °)

*D*—H⋯*A*	*D*—H	H⋯*A*	*D*⋯*A*	*D*—H⋯*A*
O1—H1⋯O2	0.90 (2)	1.77 (2)	2.611 (2)	154 (2)

**Fig. 1.**
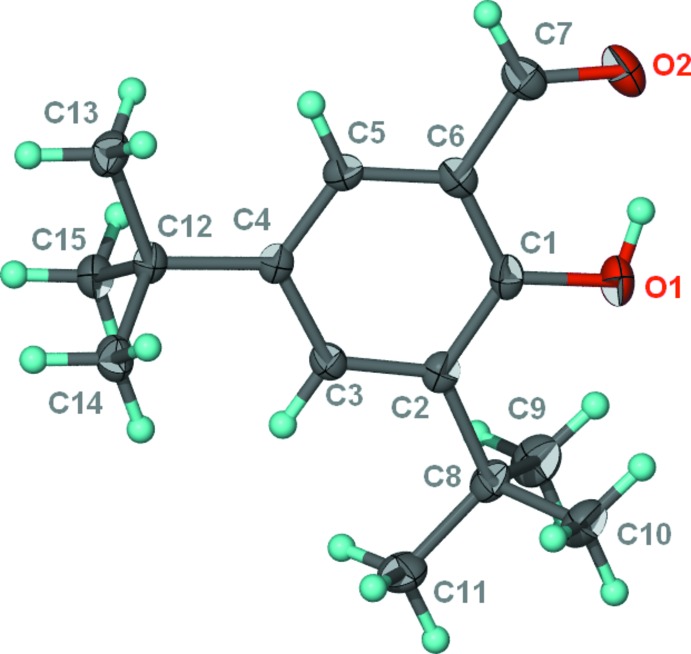
Thermal ellipsoid plot (Barbour, 2001) of (I) at the 70% probability level. Hydrogen atoms are drawn as spheres of arbitrary radius.

## supplementary crystallographic information

### 1. Comment

The title compound has been previously described in the monoclinic crystal class with two independent molecules in the asymmetric unit. In the room temperature structure, the 5-*tert*-butyl group in each molecule is disordered over two positions (Chu *et al.*, 2004) but in the structure at 150 K, in one molecule only is the 5-*tert*-butyl group disordered (Tooke & Spek, 2004). The disorder is retained even at 100 K (Ng, 2013).

In the title polymorph (I) there is one molecule in the asymmetric unit and there is no disorder. The hydroxy group forms a short intramolecular hydrogen bond with the aldehyde group. The mean plane of the six-membered hydrogen-bonded ring (O1/C1/C6/C7/O2 with maximum deviation 0.013 (1)Å for C7) is essentially co-planar with the benzene ring [dihedral angle = 2.29 (8)°]. The volume of one molecule is calculated to be 338 Å^3^ at 100 K; the volume increased marginally to 349 Å^3^ at 150 K, and at room temperature, the volume is 361 Å^3^. The absence of disorder is plausibly explained by a more efficient packing.

### 2. Experimental

3,5-Di-*tert*-butyl-2-hydroxybenzaldehyde was recovered unchanged from a reaction that used the compound as a reactant. It was recrystallized from ethanol to afford colorless primatic crystals.

### 3. Refinement

Carbon-bound H-atoms were placed in calculated positions [C–H 0.95 to 0.98 Å, *U*_iso_(H) 1.2 to 1.5*U*_eq_(C)] and were included in the refinement in the riding model approximation. The hydroxy H-atom was located in a difference Fourier map, and was freely refined.

### Crystal data

**Table:**

C_15_H_22_O_2_	*F*(000) = 512
*M**_r_* = 234.33	*D*_x_ = 1.152 Mg m^−^^3^
Monoclinic, *P*2_1_/*n*	Mo *K*α radiation, λ = 0.71073 Å
Hall symbol: -P 2yn	Cell parameters from 3060 reflections
*a* = 9.8347 (6) Å	θ = 2.9–27.5°
*b* = 11.1775 (5) Å	µ = 0.07 mm^−^^1^
*c* = 13.1287 (8) Å	*T* = 100 K
β = 110.614 (7)°	Prism, colorless
*V* = 1350.80 (13) Å^3^	0.40 × 0.30 × 0.20 mm
*Z* = 4	

### Data collection

**Table:**

Agilent SuperNova Dual diffractometer with an Atlas detector	3130 independent reflections
Radiation source: SuperNova (Mo) X-ray Source	2511 reflections with *I* > 2σ(*I*)
Mirror monochromator	*R*_int_ = 0.027
Detector resolution: 10.4041 pixels mm^-1^	θ_max_ = 27.6°, θ_min_ = 2.9°
ω scan	*h* = −12→9
Absorption correction: multi-scan (*CrysAlis PRO*; Agilent, 2013)	*k* = −14→14
*T*_min_ = 0.971, *T*_max_ = 0.985	*l* = −12→17
7536 measured reflections	

### Refinement

**Table:**

Refinement on *F*^2^	Primary atom site location: structure-invariant direct methods
Least-squares matrix: full	Secondary atom site location: difference Fourier map
*R*[*F*^2^ > 2σ(*F*^2^)] = 0.046	Hydrogen site location: inferred from neighbouring sites
*wR*(*F*^2^) = 0.119	H atoms treated by a mixture of independent and constrained refinement
*S* = 1.02	*w* = 1/[σ^2^(*F*_o_^2^) + (0.0519*P*)^2^ + 0.4143*P*] where *P* = (*F*_o_^2^ + 2*F*_c_^2^)/3
3130 reflections	(Δ/σ)_max_ = 0.001
158 parameters	Δρ_max_ = 0.29 e Å^−^^3^
0 restraints	Δρ_min_ = −0.23 e Å^−^^3^

### Fractional atomic coordinates and isotropic or equivalent isotropic displacement parameters (Å^2^)

**Table:**

	*x*	*y*	*z*	*U*_iso_*/*U*_eq_	
O1	0.82116 (10)	0.43175 (9)	0.47510 (8)	0.0249 (2)	
H1	0.833 (2)	0.489 (2)	0.5258 (17)	0.059 (6)*	
O2	0.77316 (11)	0.61059 (9)	0.58586 (8)	0.0264 (3)	
C1	0.68674 (14)	0.45184 (11)	0.40121 (10)	0.0170 (3)	
C2	0.63560 (14)	0.38221 (11)	0.30569 (10)	0.0161 (3)	
C3	0.49801 (14)	0.40954 (11)	0.23303 (10)	0.0158 (3)	
H3	0.4623	0.3636	0.1681	0.019*	
C4	0.40701 (13)	0.50021 (11)	0.24795 (10)	0.0149 (3)	
C5	0.46075 (14)	0.56620 (11)	0.34210 (10)	0.0159 (3)	
H5	0.4032	0.6284	0.3553	0.019*	
C6	0.59895 (14)	0.54334 (11)	0.41894 (10)	0.0171 (3)	
C7	0.65209 (15)	0.61768 (12)	0.51539 (11)	0.0210 (3)	
H7	0.5880	0.6767	0.5248	0.025*	
C8	0.72960 (14)	0.28431 (12)	0.28098 (11)	0.0196 (3)	
C9	0.86554 (15)	0.34226 (14)	0.26966 (13)	0.0274 (3)	
H9A	0.8360	0.4023	0.2115	0.041*	
H9B	0.9244	0.2806	0.2518	0.041*	
H9C	0.9228	0.3808	0.3384	0.041*	
C10	0.77506 (16)	0.19013 (12)	0.37206 (12)	0.0263 (3)	
H10A	0.8299	0.2289	0.4413	0.040*	
H10B	0.8361	0.1295	0.3554	0.040*	
H10C	0.6882	0.1519	0.3776	0.040*	
C11	0.64704 (16)	0.21922 (13)	0.17455 (12)	0.0260 (3)	
H11A	0.6165	0.2771	0.1146	0.039*	
H11B	0.5613	0.1798	0.1808	0.039*	
H11C	0.7105	0.1591	0.1601	0.039*	
C12	0.26001 (14)	0.52301 (11)	0.15867 (10)	0.0169 (3)	
C13	0.17026 (15)	0.61558 (12)	0.19394 (12)	0.0226 (3)	
H13A	0.2249	0.6906	0.2131	0.034*	
H13B	0.1501	0.5853	0.2572	0.034*	
H13C	0.0784	0.6299	0.1340	0.034*	
C14	0.17131 (14)	0.40643 (12)	0.13060 (11)	0.0214 (3)	
H14A	0.2270	0.3452	0.1089	0.032*	
H14B	0.0797	0.4212	0.0705	0.032*	
H14C	0.1507	0.3787	0.1945	0.032*	
C15	0.28685 (15)	0.57049 (12)	0.05752 (11)	0.0206 (3)	
H15A	0.3443	0.6442	0.0762	0.031*	
H15B	0.1935	0.5872	−0.0001	0.031*	
H15C	0.3399	0.5103	0.0319	0.031*	

### Atomic displacement parameters (Å^2^)

**Table:**

	*U*^11^	*U*^22^	*U*^33^	*U*^12^	*U*^13^	*U*^23^
O1	0.0165 (5)	0.0293 (5)	0.0222 (5)	0.0014 (4)	−0.0015 (4)	0.0000 (4)
O2	0.0243 (6)	0.0312 (6)	0.0194 (5)	−0.0085 (4)	0.0023 (4)	−0.0035 (4)
C1	0.0138 (6)	0.0188 (6)	0.0165 (6)	−0.0017 (5)	0.0031 (5)	0.0046 (5)
C2	0.0145 (6)	0.0169 (6)	0.0179 (7)	−0.0006 (5)	0.0068 (5)	0.0028 (5)
C3	0.0169 (6)	0.0164 (6)	0.0143 (6)	−0.0015 (5)	0.0054 (5)	−0.0005 (5)
C4	0.0134 (6)	0.0156 (6)	0.0157 (6)	−0.0004 (5)	0.0054 (5)	0.0028 (5)
C5	0.0165 (6)	0.0144 (6)	0.0182 (6)	−0.0007 (5)	0.0078 (5)	0.0005 (5)
C6	0.0192 (7)	0.0167 (6)	0.0155 (6)	−0.0038 (5)	0.0064 (5)	0.0006 (5)
C7	0.0229 (7)	0.0209 (7)	0.0194 (7)	−0.0055 (5)	0.0078 (6)	−0.0015 (6)
C8	0.0146 (7)	0.0198 (6)	0.0248 (7)	0.0030 (5)	0.0075 (6)	0.0019 (6)
C9	0.0183 (7)	0.0314 (8)	0.0359 (9)	0.0024 (6)	0.0140 (6)	0.0016 (7)
C10	0.0247 (8)	0.0221 (7)	0.0323 (8)	0.0061 (6)	0.0100 (6)	0.0055 (6)
C11	0.0238 (8)	0.0255 (7)	0.0301 (8)	0.0054 (6)	0.0114 (6)	−0.0053 (6)
C12	0.0134 (6)	0.0188 (6)	0.0168 (6)	0.0010 (5)	0.0033 (5)	0.0013 (5)
C13	0.0178 (7)	0.0259 (7)	0.0233 (7)	0.0056 (5)	0.0060 (6)	0.0014 (6)
C14	0.0155 (7)	0.0232 (7)	0.0220 (7)	−0.0017 (5)	0.0024 (5)	0.0006 (6)
C15	0.0192 (7)	0.0219 (7)	0.0179 (7)	0.0014 (5)	0.0031 (5)	0.0009 (5)

### Geometric parameters (Å, º)

**Table:**

O1—C1	1.3551 (15)	C9—H9C	0.9800
O1—H1	0.90 (2)	C10—H10A	0.9800
O2—C7	1.2265 (17)	C10—H10B	0.9800
C1—C2	1.4097 (18)	C10—H10C	0.9800
C1—C6	1.4096 (18)	C11—H11A	0.9800
C2—C3	1.3877 (17)	C11—H11B	0.9800
C2—C8	1.5390 (17)	C11—H11C	0.9800
C3—C4	1.4106 (17)	C12—C13	1.5336 (18)
C3—H3	0.9500	C12—C15	1.5367 (18)
C4—C5	1.3751 (18)	C12—C14	1.5389 (18)
C4—C12	1.5283 (17)	C13—H13A	0.9800
C5—C6	1.4018 (18)	C13—H13B	0.9800
C5—H5	0.9500	C13—H13C	0.9800
C6—C7	1.4494 (18)	C14—H14A	0.9800
C7—H7	0.9500	C14—H14B	0.9800
C8—C11	1.5304 (19)	C14—H14C	0.9800
C8—C10	1.5368 (19)	C15—H15A	0.9800
C8—C9	1.5391 (18)	C15—H15B	0.9800
C9—H9A	0.9800	C15—H15C	0.9800
C9—H9B	0.9800		
			
C1—O1—H1	104.6 (14)	C8—C10—H10B	109.5
O1—C1—C2	119.86 (12)	H10A—C10—H10B	109.5
O1—C1—C6	120.13 (12)	C8—C10—H10C	109.5
C2—C1—C6	120.01 (12)	H10A—C10—H10C	109.5
C3—C2—C1	116.49 (11)	H10B—C10—H10C	109.5
C3—C2—C8	121.63 (11)	C8—C11—H11A	109.5
C1—C2—C8	121.84 (11)	C8—C11—H11B	109.5
C2—C3—C4	125.14 (12)	H11A—C11—H11B	109.5
C2—C3—H3	117.4	C8—C11—H11C	109.5
C4—C3—H3	117.4	H11A—C11—H11C	109.5
C5—C4—C3	116.63 (12)	H11B—C11—H11C	109.5
C5—C4—C12	124.09 (11)	C4—C12—C13	111.74 (11)
C3—C4—C12	119.23 (11)	C4—C12—C15	108.45 (10)
C4—C5—C6	121.17 (12)	C13—C12—C15	108.67 (11)
C4—C5—H5	119.4	C4—C12—C14	110.24 (10)
C6—C5—H5	119.4	C13—C12—C14	107.79 (11)
C5—C6—C1	120.56 (12)	C15—C12—C14	109.93 (11)
C5—C6—C7	118.96 (12)	C12—C13—H13A	109.5
C1—C6—C7	120.45 (12)	C12—C13—H13B	109.5
O2—C7—C6	125.29 (13)	H13A—C13—H13B	109.5
O2—C7—H7	117.4	C12—C13—H13C	109.5
C6—C7—H7	117.4	H13A—C13—H13C	109.5
C11—C8—C10	107.47 (11)	H13B—C13—H13C	109.5
C11—C8—C9	108.16 (11)	C12—C14—H14A	109.5
C10—C8—C9	109.80 (11)	C12—C14—H14B	109.5
C11—C8—C2	111.44 (11)	H14A—C14—H14B	109.5
C10—C8—C2	110.78 (11)	C12—C14—H14C	109.5
C9—C8—C2	109.13 (11)	H14A—C14—H14C	109.5
C8—C9—H9A	109.5	H14B—C14—H14C	109.5
C8—C9—H9B	109.5	C12—C15—H15A	109.5
H9A—C9—H9B	109.5	C12—C15—H15B	109.5
C8—C9—H9C	109.5	H15A—C15—H15B	109.5
H9A—C9—H9C	109.5	C12—C15—H15C	109.5
H9B—C9—H9C	109.5	H15A—C15—H15C	109.5
C8—C10—H10A	109.5	H15B—C15—H15C	109.5
			
O1—C1—C2—C3	−178.86 (11)	C2—C1—C6—C7	−178.25 (11)
C6—C1—C2—C3	0.20 (18)	C5—C6—C7—O2	−176.38 (13)
O1—C1—C2—C8	−1.20 (18)	C1—C6—C7—O2	1.7 (2)
C6—C1—C2—C8	177.85 (11)	C3—C2—C8—C11	−4.45 (17)
C1—C2—C3—C4	−0.27 (19)	C1—C2—C8—C11	178.02 (12)
C8—C2—C3—C4	−177.93 (11)	C3—C2—C8—C10	−124.05 (13)
C2—C3—C4—C5	0.29 (19)	C1—C2—C8—C10	58.41 (16)
C2—C3—C4—C12	177.79 (11)	C3—C2—C8—C9	114.93 (13)
C3—C4—C5—C6	−0.23 (18)	C1—C2—C8—C9	−62.60 (15)
C12—C4—C5—C6	−177.60 (11)	C5—C4—C12—C13	−8.45 (17)
C4—C5—C6—C1	0.18 (19)	C3—C4—C12—C13	174.25 (11)
C4—C5—C6—C7	178.29 (11)	C5—C4—C12—C15	111.31 (13)
O1—C1—C6—C5	178.89 (11)	C3—C4—C12—C15	−65.99 (14)
C2—C1—C6—C5	−0.16 (18)	C5—C4—C12—C14	−128.29 (13)
O1—C1—C6—C7	0.80 (18)	C3—C4—C12—C14	54.40 (15)

### Hydrogen-bond geometry (Å, º)

**Table:**

*D*—H···*A*	*D*—H	H···*A*	*D*···*A*	*D*—H···*A*
O1—H1···O2	0.90 (2)	1.77 (2)	2.611 (2)	154 (2)
